# Assessing the Effects of a Diet of BPA Analogue-Exposed Microalgae in the Clam *Ruditapes philippinarum*

**DOI:** 10.3390/jox14030069

**Published:** 2024-09-06

**Authors:** Jacopo Fabrello, Michela Dalla Fontana, Noemi Gaiani, Maria Ciscato, Marco Roverso, Sara Bogialli, Valerio Matozzo

**Affiliations:** 1 Department of Biology, University of Padova, Via Bassi 58/B, 35131 Padova, Italy; michela.dallafontana@studenti.unipd.it (M.D.F.); noemi.gaiani@studenti.unipd.it (N.G.); mariaciscato95@gmail.com (M.C.); 2 Department of Chemical Sciences, University of Padova, Via Marzolo 1, 35131 Padova, Italy; marco.roverso@unipd.it (M.R.); sara.bogialli@unipd.it (S.B.)

**Keywords:** bisphenol A analogues, microalgae, clams, dietary exposure, biomarkers, bioaccumulation, antioxidants

## Abstract

In our previous study, we demonstrated that the microalgae *Phaeodactylum tricornutum* can bioaccumulate bisphenol A analogues. Since this microalgae species is part of the diet of marine filter-feeding organisms, such as bivalves, in this study we tested the hypothesis that a diet based on exposed microalgae can exert negative effects on the clam *Ruditapes philippinarum*. Microalgae were exposed for 7 days to 300 ng/L of bisphenol AF (BPAF), bisphenol F (BPF), and bisphenol S (BPS), alone or as a mixture (MIX), to allow bioaccumulation. Microalgae were then supplied as food to bivalves. After 7 and 14 days of diet, the effects of exposed microalgae were evaluated on a battery of biomarkers measured in haemolymph/haemocytes, gills and digestive glands of clams. In addition, bioaccumulation of the three bisphenols was investigated in clams by UHPLC-HRMS. The results obtained demonstrated that total haemocyte count (THC) increased in clams following ingestion for 7 days of BPAF- and BPF-exposed microalgae, while BPS-exposed microalgae significantly reduced THC after 14 days of diet. MIX- and BPS-exposed microalgae increased haemocyte proliferation. The diet of exposed microalgae affected acid and alkaline phosphatase activity in clams, with an opposite response between haemolymph and haemocytes. Regarding antioxidants, an increase in catalase activity was observed in clams after ingestion of BPA analogue-exposed microalgae. The results also demonstrated marked oxidative stress in gills, the first tissue playing an important role in the feeding process. Oxidative damage was recorded in both the gills and digestive glands of clams fed BPA analogue-exposed microalgae. Alterations in epigenetic-involved enzyme activity were also found, demonstrating for the first time that BPA analogue-exposed food can alter epigenetic mechanisms in marine invertebrates. No bioaccumulation of BPA analogues was detected in clam soft tissues. Overall, this study demonstrated that a diet of BPA analogue-exposed microalgae can induce significant alterations of some important biological responses of *R. philippinarum*. To our knowledge, this is the first study demonstrating the effects of ingestion of BPA analogue-exposed microalgae in the clam *R. philippinarum*, suggesting a potential ecotoxicological risk for the marine food chain, at least at the first levels.

## 1. Introduction

Bisphenol A (BPA) is the most used bisphenol worldwide, with production still growing [1]. BPA is mainly used in polycarbonate plastic production [2]. However, the increasing evidence on the estrogenic activity of BPA also in aquatic species [3] has led to some limitations in its uses. Consequently, the replacement of BPA with other similar compounds—named BPA analogues—has begun. At least 17 different native bisphenols and another 131 chemicals derived from them have been synthesised.

Three of the most known BPA analogues are bisphenol AF (BPAF), bisphenol F (BPF) and bisphenol S (BPS), being largely used to produce polycarbonate copolymers, epoxy resins, liners, water pipes, toys, adhesives, food packaging and thermal paper [4]. The increasing use of such BPA analogues led to a consequent release of detectable concentrations into ecosystems, including freshwater and seawater. Generally, environmental concentrations of BPA analogues range from few ng/L up to hundreds of ng/L in both freshwater and seawater [5]. For example, a concentration of up to 140 ng/L of BPAF was recorded in Taihu Lake in China [6]. Moreover, BPF reached an environmental concentration of up to 2850 ng/L in the Tamagawa River in Japan [7]. High levels of BPS (up to 65,600 ng/L) were recorded in rivers of China [8]. As for the marine coastal environment, the concentrations of BPA analogues are generally lower than those recorded in freshwater [5]. However, concentrations of 282 ng/L and 1470 ng/L of BPF were detected in seawater in South China and Tokyo Bay, respectively [7,9]. Furthermore, BPA was detected at a mean concentration of 13 ng/g dw (dry weight) in marine sediments from coastal areas of Zhejiang in East China [10]. In the same area, BPF, BPAF and BPS reached concentrations of 1.6 ng/g dw, 0.53 ng/g dw and 0.69 ng/g dw, respectively [10]. In that study, all seawater samples contained measurable concentrations of BPA (mean 23 ng/L, range 2.7–52 ng/L), BPS (2.2 ng/L, 0.15–12 ng/L), and BPAF (0.34 ng/L, 0.12–0.91 ng/L), while BPF was only detectable in some seawater samples at concentrations lower than 1.0 ng/L [10]. BPA was also predominant in surface seawater and sediment samples from the Beibu Gulf, South China Sea, with concentrations ranging from 5.26 to 12.04 ng/L in seawater and from 0.56 to 5.22 ng/g dw in sediment samples, followed by BPAF (0.44–0.60 ng/L in seawater and 0.08–0.66 ng/g dw in sediments, respectively) and BPS (0.07–0.63 ng/L in seawater and up to 0.19 ng/g dw in sediments, respectively) [11]. The predicted no-effect concentrations (PNECs) in freshwater and seawater for the three compounds are, respectively, 1.02 µg/L and 100 ng/L for BPAF, 5.44 µg/L and 540 ng/L for BPF, and 12.9 µg/L and 27 µg/L for BPS [12]. However, the only adopted PNEC with legislative relevance is the BPA PNEC, which was settled at 1500 ng/L for freshwater and 150 ng/L in seawater by the European Union [13].

In a recent study, we demonstrated that exposure to BPAF, BPF and BPS induced oxidative stress and ultrastructural changes in the microalgae *Phaeodactylum tricornutum* [14]. In that study, bioaccumulation of the three BPA analogues in microalgae was also assessed, and the results obtained demonstrated that BPAF and BPS were bioaccumulated in microalgae. In another study, we evaluated the effects and bioaccumulation of the same BPA analogues (at the same concentrations) in the clam *Ruditapes philippinarum* [15]. However, information regarding the toxicity of these compounds in aquatic organisms, whether exposed to water or subjected to a diet of pre-exposed food, is rather scarce. The present study tries to fill this knowledge gap. Consequently, we evaluated for the first time the effects of food-borne exposure to BPAF, BPF, and BPS—alone or as a mixture—on some important biomarkers in the clam *Ruditapes philippinarum*. The aim of this study was to compare the effects caused by the ingestion of BPA analogue-exposed microalgae to the effects observed in a recent study in which bivalves were exposed to the same contaminants dissolved in seawater [15].

## 2. Materials and Methods

### 2.1. Microalgae Culture and Exposure

*P. tricornutum* was purchased from the Culture Collection of Algae at Göttingen University (SAG). Microalgae were grown for 10 days in F/2 medium [16] prepared in 0.45 μm filtered seawater at 16 °C, with a light intensity corresponding to 40.5 µmol photons m^−2^s^−1^, and a photoperiod of 12:12 light/dark. BPAF and BPF stock solutions (1 mg/L) were prepared in methanol, while the BPS solution (1 mg/L) was prepared in distilled water. Five experimental conditions, namely control, BPAF, BPF, BPS and their mixture (MIX), were prepared in Erlenmeyer flasks with an F/2 volume of 600 mL at an initial concentration of microalgae of 5 × 10^5^ cells/mL (*inoculum*). BPA analogues were added in the corresponding experimental condition at a final concentration of 300 ng/L. As for the mixture, microalgae were exposed to 100 ng/L of each compound. We chose these concentrations because they are in the same order of magnitude as the BPA analogue concentrations recorded in marine coastal areas.

Microalgae were treated for 7 days to allow them to bioaccumulate BPA analogues [14]. A solvent control was not performed because we previously observed that methanol does not cause negative effects on microalgae [14]. In addition, it has been demonstrated that methanol can cause toxic effects at very high concentrations in aquatic species (tens and hundreds of mg/L), including marine microalgae [17,18].

### 2.2. Clam Acclimation and Treatment

*R. philippinarum* specimens were sampled in February 2023 from a licenced fishing area in the Lagoon of Venice (Italy). Then, molluscs were acclimated in large aquaria filled with aerated seawater (salinity of 35 ± 1, temperature of 11 ± 0.5 °C) and a sandy bottom for 7 days. After acclimation, 80 clams (mean length: 36.7 mm) were randomly divided into 10 experimental tanks without sand (30-litre capacity, 2 tanks per experimental condition, 40 clams per tank). Every two days, seawater was renewed, and 200 mL of control or exposed microalgae suspensions were added. To allow clams to take up contaminants only from the microalgae and not from the medium in which they grew, all microalgae suspensions (both control and treated groups) were centrifuged at 4000× *g* rpm at room temperature for 10 min using an ultracentrifuge Avant-J-25. The supernatant (=BPA analogue-exposed medium) was discharged, and microalgae were then carefully re-suspended in 0.45 mm filtered seawater. Clam tissues were collected after 7 and 14 days of diet with control or exposed microalgae.

### 2.3. Tissue Collection

A 1 mL syringe was used to collect the haemolymph from the anterior adductor muscle of clams. We prepared 5 pools of haemolymph (from six clams each) for each experimental condition at each tissue sampling time (7 and 14 days). After sampling, total haemocyte count (THC), haemocyte diameter and volume, lactate dehydrogenase (LDH) activity and haemocyte proliferation (XTT assay) were measured. To obtain cell-free haemolymph (CFH) and haemocyte lysate (HL), pooled haemolymph was centrifuged at 780× *g* for 10 min, the pellets (=haemocytes) were then re-suspended in distilled water to obtain HL, whereas supernatants (CFH) were collected and stored on ice. Both CFH and HL samples were frozen in liquid nitrogen and stored at −80 °C until analyses. After haemolymph sampling, gills and digestive glands were excised and pooled (five pools of six clams each). Aliquots of each pooled tissue were then frozen in liquid nitrogen and stored at −80 °C until analyses.

### 2.4. Haemolymph and Haemocyte Biomarkers

A Scepter™ 2.0 Automated Cell Counter (Millipore, FL, USA) was used to determine the THC, as well as the haemocyte diameter and volume. In detail, 20 μL of haemolymph was diluted into 2 mL of Coulter Isoton II diluent. THC was expressed as the number of haemocytes (10^5^)/mL of haemolymph, while haemocyte diameter and volume were expressed in μm and picolitres (pL), respectively.

Cell-free haemolymph (CFH) LDH activity was measured using the commercial kit *Cytotoxicity Detection* Kit (Roche). Briefly, after centrifugation (780× *g* for 10 min), 500 μL of CFH was mixed with an equal volume of reagent provided with the kit. After 30 min, we measured the absorbance, and the results were expressed as optical density (OD) at 490 nm.

To evaluate haemocyte proliferation, we used the *Cell proliferation* Kit II. In detail, a volume of the reagent mixture (provided with the kit) was added to two volumes of pooled haemolymph and incubated for 4 h at room temperature. Then, we measured the absorbance at 450 nm and the results were normalised to THC values of each sample and expressed as optical density (OD) at 450 nm.

Lysozyme activity was measured in haemocyte lysate (HL) by mixing 50 μL of HL with 950 μL of a 0.15% suspension of *Micrococcus lysodeikticus* (Sigma) phosphate buffer (pH 6.2). The decrease in absorbance was recorded for 3 min at 450 nm at room temperature. Results were expressed as μg lysozyme/mg of proteins.

The arylsulfatase activity was measured in HL samples measuring the production of p-nitrocatechol after 1 h at 515 nm [19] and then calculated using the formula proposed by Baum et al. [20]. Results are expressed as μg of p-nitrocatechol produced per hour/mg of proteins.

The acid phosphatase and alkaline phosphatase activity were measured both in HL and CFH. The acid phosphatase hydrolysed the substrate 4-nitrophenyl phosphate during the incubation at 37 °C and the absorbance was measured at 405 nm using a microplate reader. Results were expressed as U/mg of proteins. Similarly, the alkaline phosphatase hydrolysed the same substrate in an alkaline buffer, and after the incubation at 30 °C, the absorbance was recorded at 405 nm [21].

Lastly, the total antioxidant capacity of haemolymph was assessed following the cupric reducing antioxidant capacity (CUPRAC) method [22]. In detail, the cupric ions produced a coloured complex with neocuproine. Then, the absorbance was measured at 450 nm using a microplate reader. Results are reported as mM of Trolox equivalents/mg of proteins.

### 2.5. Gill and Digestive Gland Biomarkers

Gills and digestive gland samples were homogenised using the TissueLyser LT (Qiagen). In detail, samples were homogenised in four volumes of 10 mM Tris-HCl buffer, pH 7.5, containing 0.15 M KCl, 0.5 M sucrose, 1 mM EDTA and protease inhibitor cocktail (1:10 *v*/*v*) (Merck, Milano, Italy) at 50 oscillations per second for 5 min at 4 °C. The samples were centrifuged at 12,000× *g* for 30 min at 4 °C and supernatants (SNs) were collected for analyses. All analyses were performed in triplicate.

Like haemolymph, a CUPRAC assay was performed in SNs of both gills and digestive glands according to the CUPRAC method [22]. The results were expressed as mM of Trolox equivalents/mg of proteins.

Total superoxide dismutase (SOD) activity was measured following the xanthine oxidase/cytochrome *c* method in both gill and digestive gland SNs [23]. Enzyme activity was expressed as U/mg proteins, and one unit of SOD has been defined as the amount of sample causing 50% inhibition under the assay conditions.

Catalase (CAT) activity was measured in gill and digestive gland SNs by recording the absorbance at 240 nm. Results were expressed as U/mg proteins [24]. One unit of CAT was defined as the amount of enzyme that catalysed the dismutation of 1 μmol of H_2_O_2_/min.

Acetylcholinesterase (AChE) activity was measured only in gills following the colorimetric reaction between acetylthiocholine and the reagent dithiobisnitrobenzoate [25]. The increase in absorbance at 405 nm was recorded for 5 min using a microplate reader at room temperature. Results are expressed as nmol/min/mg of protein. Similarly, the butyrylcholinesterase (BChE) activity was measured using butyrylthiocholine as a substrate and the absorbance was quantified at 405 nm [26]. The enzymatic activity is expressed as nmol/min/mg proteins.

Glutathione reductase (GR) activity was measured in both gill and digestive gland SNs following the method proposed by Smith et al. [27]. In detail, we quantified the amount of 5-thio (2-nitrobenzoic acid) produced at 412 nm. Results are expressed as U/mg proteins.

Glutathione S-transferase (GST) activity was evaluated only in the digestive gland SN using 1-chloro-2,4-dinitrobenzene (CDNB) and reduced glutathione (GSH) as substrates [28]. GST activity was expressed as nmol/min/mg proteins.

The protein carbonyl content (PCC) and lipid peroxidation (LPO) were measured as oxidative damage biomarkers. Briefly, PCC was measured using the method of Mecocci et al. [29]. This spectrophotometric method is based on the reaction of carbonyl groups with 2,4-dinitrophenylhydrazine (DNPH). Results were expressed as nmol carbonyl group/mg of proteins.

LPO was quantified according to the method of Buege and Aust [30]. The method is based on the quantification of malondialdehyde (MDA) at 532 nm and the results were expressed as nmoles of thiobarbituric reactive substances (TBARSs)/mg of proteins. TBARSs, considered as “MDA-like peroxide products”, were quantified by reference to MDA absorbance (ε = 156 × 10^3^ M^−1^ cm^−1^) [31].

Total protein concentration in SN samples was quantified according to Bradford et al. [32].

### 2.6. Epigenetic Biomarkers

The histone N-terminal acetyltransferases (HATs) and histone deacetylases activities (HDACs) were evaluated in gills and digestive glands. The HAT activity was measured at 412 nm [33]. In detail, SN samples were prepared as described above, followed by a sonication step. Then, we used the histone extracted from the calf thymus (Sigma Aldrich, Milan, Italy) as an acetyl acceptor and acetyl-CoA as an acetyl group donator. The resulting free thiol group was quantified using 5,5′-dithiobis-(2-nitrobenzoic acid) at 412 nm in a microplate. Results are expressed as μM of 5-thio-2-nitrobenzoic acid (TNB^-^)/mg of proteins. The HDAC activity of class I and II was quantified following the spectrophotometric method proposed by Yuan et al. [34]. Briefly, SN samples reacted with the synthetic substrate Boc-Lys(Ac)-pNA, removing the acetyl group from the lysine. This reaction led to the formation of a chromogen compound that was quantified at 405 nm in a microplate. Results are expressed as OD at 405 nm/mg of proteins.

### 2.7. Bioaccumulation

Methanol, acetonitrile, ammonium acetate, BPAF, BPF, BPS and bisphenol A d-16, used as internal standard, were purchased from Merck (Milan, Italy), while the ultrapure-grade water was produced with a Pure-Lab Option Q apparatus (Elga Lab Water, High Wycombe, UK). Bioaccumulation of BPs was evaluated in five organisms collected after 7 and 14 days of diet with BPA analogue-exposed microalgae. Each sample was accurately weighed and homogenised (Homogeniser SHM1, Avantor, VWR International Srl, Milano, Italia) after the addition of 1 mL of ultrapure water. The homogenate was treated with cold acetonitrile (7 mL) containing the internal standard at 500 µg/L, vortexed for 3 min, and centrifuged at 5000× *g* rpm for 5 min. After a further centrifugation step (13,000× *g* rpm, 10 min, 4 °C), 20 µL of the supernatant was analysed by UHPLC-HRMS. The system was equipped with an Agilent 1260 Infinity II LC chromatographer coupled to an Agilent 6545 LC/Q-TOF mass analyser (Agilent Technologies, Palo Alto, Santa Clara, CA, USA). The analytical column was a Kinetex 2.6 µm C18 Polar, 100 A, 100 × 2.1 mm (Phenomenex, Bologna, Italy), at 25 °C. Mobile phases A and B were water and acetonitrile, respectively, both containing 10 mM ammonium acetate, and the eluent flow rate was 0.30 mL/min. The mobile-phase gradient profile was as follows (t in min): t0–4 0% B; t4–22 0–100% B, t22–25 100% B; t25–32 0% B. The MS conditions were electrospray (ESI) ionisation in negative mode, gas temperature of 320 °C, drying gas at 12 L/min, nebulizer at 35 psi, sheath gas temperature of 350 °C, sheath gas flow of 11 L/min, VCap at 5000 V, nozzle voltage of 0 V, and fragmentor at 150 V. Centroid full-scan mass spectra were recorded in the range 100–1000 *m*/*z* with a scan rate of 2 spectra/s. The QTOF calibration was performed daily with the manufacturer’s solution in this mass range. The Mass Hunter Qualitative Analysis software (Agilent Technologies, Palo Alto, Santa Clara, CA, USA) was used to analyse the MS.

Homogenates from untreated organisms were used to build a matrix-matched seven-point external calibration curve, in the range 0.1–100 µg/L (corresponding to 0.8–800 ng/g in the initial animal tissues). Linearity was evaluated by least squares regression and R^2^ > 0.998 was obtained for all the analytes. LODs were 40 ng/g for BPF, 2 ng/g for BPS and 1 ng/g for BPAF. Each treated organism was analysed separately, and results are reported as mean and standard deviation.

### 2.8. Statistical Analysis

The normal distribution of data (Shapiro–Wilk’s test) and the homogeneity of the variances (Bartlett’s test) were assessed. The results obtained were compared by performing the two-way ANOVA analysis, using “exposure time”, “treatment” (=diet) and “exposure time–treatment interaction” as independent factors. Pairwise comparisons among experimental conditions were performed using Fisher’s LSD post hoc test. The significant difference was set at *p* < 0.05. All results are expressed as means ± standard deviation (SD), n = 5. The software package Origin 2023 (Origin Lab, Northampton, MA, USA) was used for statistical analyses.

## 3. Results

### 3.1. Haemolymph and Haemocyte Biomarkers

**Figure 1:**
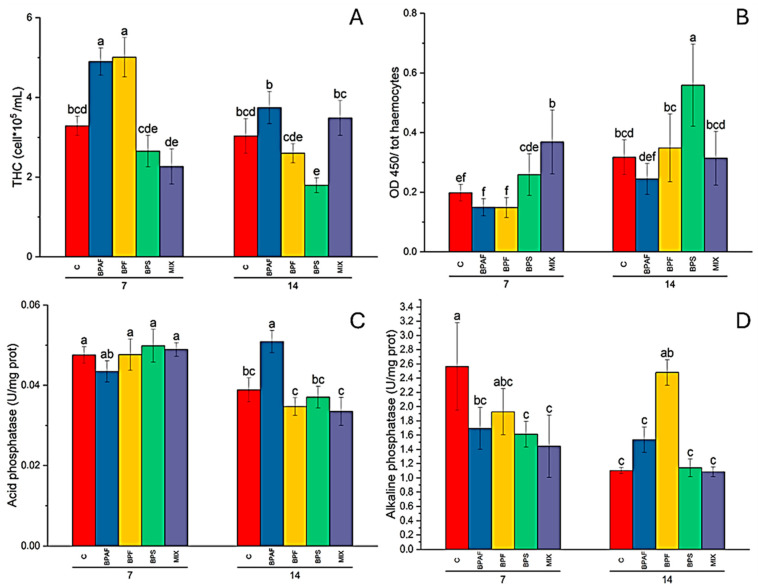
Total haemocyte count (THC), expressed as n° haemocytes (10^5^)/mL haemolymph (**A**); haemocyte proliferation, expressed as OD450 (**B**); acid phosphatase activity in CFH, expressed as U/mg proteins (**C**); and alkaline phosphatase activity in HL, expressed as U/mg proteins (**D**), in clams fed with bisphenol-exposed microalgae. Different letters indicate significant differences among all treatments. N = 5.

The two-way ANOVA analysis demonstrated that the factors “exposure time” (two-way ANOVA: *p* < 0.01), “treatment” (two-way ANOVA: *p* < 0.001) and “exposure time–treatment interaction” (two-way ANOVA: *p* < 0.001) significantly affected THC in clams. The post hoc test revealed a significant increase in THC values in clams fed for 7 days with BPAF- and BPF-treated microalgae (Figure 1A). Moreover, clams fed with BPS-exposed microalgae showed a significant reduction in THC at 14 days when compared to the related control (Figure 1A).

No significant alterations in both the diameter and volume of haemocytes were observed (Figure S1 and Figure S2 in Supplementary Material, respectively).

Similarly, ingestion of BPA analogue-exposed microalgae did not induce cytotoxicity (LDH assay) in clam haemocytes (Figure S3).

On the contrary, cell proliferation was significantly affected by all the independent factors (two-way ANOVA: *p* < 0.001). Pairwise comparisons revealed a significant increase in haemocyte proliferation in clams fed for 7 days with MIX-exposed microalgae and in those fed for 14 days with BPS-treated microalgae, with respect to the related controls (Figure 1B).

Regarding immune-related enzyme activity, only a significant (two-way ANOVA: *p* < 0.05) effect of the factor “exposure time” on both lysozyme and arylsulfatase activity was observed (Figure S4 and Figure S5, respectively). As for acid phosphatase and alkaline phosphatase activity, a different response of HL and CFH was recorded. Indeed, HL acid phosphatase activity was influenced only by the factor “exposure time” (two-way ANOVA: *p* < 0.05) (Figure S6), while the factors “exposure time” (two-way ANOVA: *p* < 0.001) and its interaction with treatment (two-way ANOVA: *p* < 0.01) had a significant effect in CFH enzyme activity. A post hoc test revealed a significant increase in CFH acid phosphatase activity in clams fed for 14 days with BPAF-exposed microalgae, when compared to the related control (Figure 1C). As for alkaline phosphatase activity, no significant alteration was found in CFH (Figure S7), while all the independent factors affected (two-way ANOVA: *p* < 0.05) enzyme activity. In the latter case, the pairwise comparisons revealed a significant reduction in enzyme activity in clams fed for 7 days with microalgae treated with BPAF, BPS and MIX, whereas significantly increased enzyme activity was observed in clams fed with BPF-treated microalgae, with respect to the related controls (Figure 1D).

Lastly, no significant effects of exposed microalgae on the total antioxidant capacity of haemolymph (assessed using CUPRAC assay) were observed (Figure S8).

### 3.2. Gill and Digestive Gland Biomarkers

**Figure 2:**
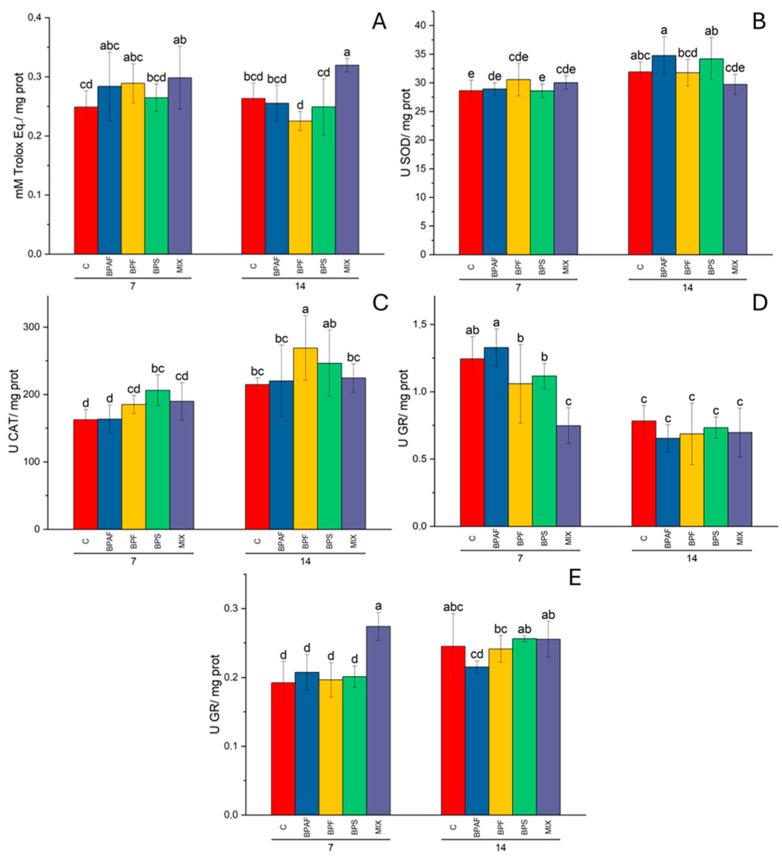
Gill CUPRAC (**A**), expressed as mM of Trolox equivalents/mg proteins; digestive gland SOD activity (**B**), expressed as U/mg proteins; gill CAT activity (**C**), expressed as U/mg proteins; and GR activity in digestive glands (**D**) and gills (**E**), expressed as U/mg proteins, in clams fed with bisphenol-exposed microalgae. Different letters indicate significant differences among all treatments. N = 5.

In gills, the factor “treatment” significantly altered (two-way ANOVA: *p* < 0.01) the CUPRAC levels, and the post hoc test revealed significantly increased CUPRAC levels in clams fed for 7 and 14 days with microalgae treated with MIX (Figure 2A). On the contrary, digestive gland CUPRAC levels were altered by the factor “exposure time” (two-way ANOVA: *p* < 0.001) (Figure S9).

Total SOD activity was influenced by the factor “exposure time” (two-way ANOVA: *p* < 0.01) in gills (Figure S10), while the factors “exposure time” (two-way ANOVA: *p* < 0.001) and its interaction with treatment (two-way ANOVA: *p* < 0.05) influenced SOD activity in digestive glands significantly, even if the post hoc test did not reveal significant differences among experimental conditions (Figure 2B).

Two-way ANOVA revealed that the factors “exposure time” (*p* < 0.001) and “treatment” (*p* < 0.05) affected CAT activity in gills, with a significant increase in enzyme activity recorded in clams fed for 7 days with microalgae treated with BPS and in those fed for 14 days with BPF-treated microalgae (Figure 2C). Only the factor “exposure time” had a significant effect (two-way ANOVA: *p* < 0.01) on CAT activity in the digestive gland (Figure S11).

As for GR activity, two-way ANOVA demonstrated that all the independent factors affected enzyme activity in both gills (two-way ANOVA: *p* < 0.001, for the factors exposure time and treatment; *p* < 0.01 for the factor exposure time–treatment interaction) and digestive glands (two-way ANOVA: *p* < 0.001, for the factor exposure time; *p* < 0.01 for the factors treatment and exposure time–treatment interaction). The post hoc test revealed a significant decrease in GR activity in digestive glands in clams fed for 7 days with MIX-treated microalgae, when compared to the related control (Figure 2D). Significantly increased GR activity was observed in gills of clams fed for 7 days with MIX-exposed microalgae, with respect to the related control (Figure 2E).

Only the factor “exposure time” significantly affected digestive gland GST activity (two-way ANOVA: *p* < 0.05) (Figure S12).

**Figure 3:**
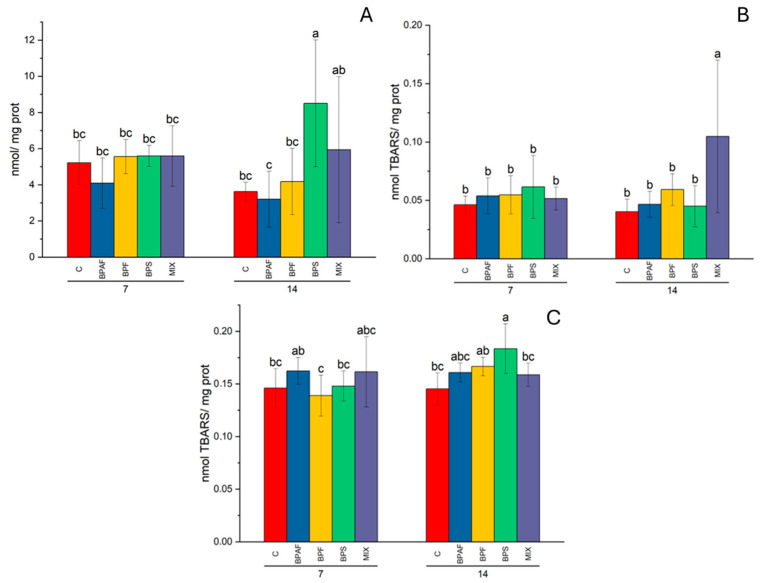
PCC levels in digestive gland (**A**), expressed as nmol/mg proteins, and LPO levels in digestive gland (**B**) and in gills (**C**), expressed as nmol TBARS/mg proteins, in clams fed with bisphenol-exposed microalgae. Different letters indicate significant differences among all treatments. N = 5.

As for oxidative damage biomarkers, namely PCC and LPO levels, significant effects of experimental conditions were observed. The PCC level was influenced by the factors “exposure time” in gills (two-way ANOVA: *p* < 0.001) (Figure S13) and “treatment” (two-way ANOVA: *p* < 0.01) in the digestive gland. In the latter tissue, significantly increased PCC levels were observed in clams fed for 14 days with BPS-treated microalgae (Figure 3A). In the digestive gland, LPO levels were influenced by the factors “treatment” and its interaction with exposure time (two-way ANOVA: *p* < 0.05), and the post hoc test highlighted a significant increase in LPO in clams fed for 14 days with MIX- treated microalgae (Figure 3B). In gills, LPO values were influenced by the factors “exposure time” and “exposure time–treatment interaction” (two-way ANOVA: *p* < 0.05). The post hoc test demonstrated that BPS-exposed microalgae caused an increase in LPO levels in the clams fed for 14 days (Figure 3C).

Regarding neurotoxicity biomarkers, only the factor “exposure time” affected gill AChE activity (two-way ANOVA: *p* < 0.01), while all the independent factors did not affect gill BChE activity (Figure S14 and Figure S15, respectively).

### 3.3. Epigenetic Biomarkers

**Figure 4:**
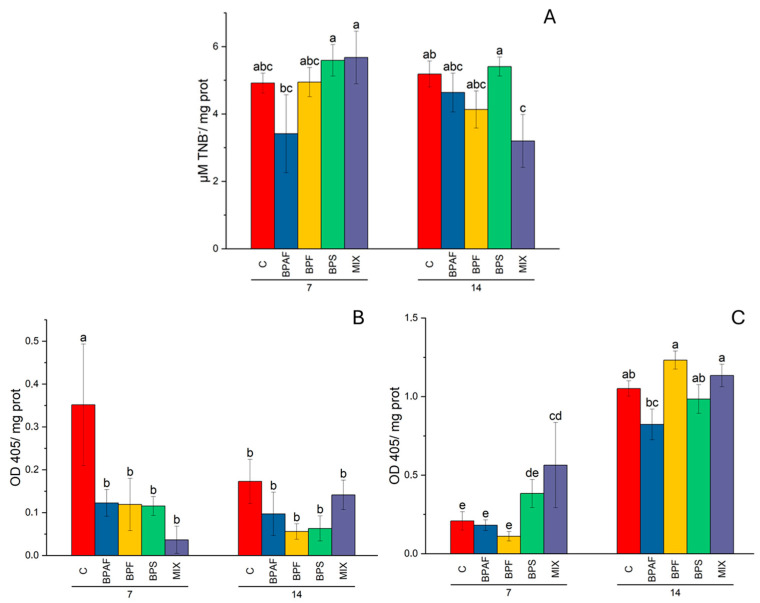
Histone acetyltransferase in digestive glands (**A**), expressed as µmol TNB^-^/mg proteins, and histone deacetylase in gills (**B**) and in digestive glands (**C**), expressed as OD405/ mg proteins. Different letters indicate significant differences among all treatments. N = 5.

The HAT activity was measured in clam gills and digestive glands. In the first tissue, independent factors did not affect HAT according to the two-way ANOVA analysis (Figure S16), while in the digestive glands, the factor “exposure time*treatment interaction” significantly affected this epigenetic-involved enzyme activity (*p* < 0.05). The post hoc test showed that clams fed for 14 days with microalgae exposed to MIX had significantly reduced HAT activity (Figure 4A). Regarding the HDAC activity, the factor “treatment” significantly altered enzyme activity in gills (*p* < 0.05). Interestingly, the post hoc test indicated that all clams fed with exposed microalgae had significantly reduced enzyme activity after 7 days of diet (Figure 4B). As for the digestive gland, HDAC activity was altered by the factors “exposure time” (*p* < 0.001) and “treatment” (*p* < 0.05). In particular, clams fed MIX-exposed microalgae showed significantly increased HDAC activity after 7 days (Figure 4C).

### 3.4. Bioaccumulation

Chemical analyses have shown that clams fed for 7 or 14 days with exposed microalgae did not bioaccumulate the three bisphenols. Indeed, measured concentrations were always <LOD for all the samples analysed.

## 4. Discussion

To the best of our knowledge, this is the first study demonstrating the effects of a diet of BPA analogue-exposed microalgae in the clam *R. philippinarum*. Consequently, the comparison of our results with those of the literature is limited and often refers to data obtained in aquatic organisms exposed to water contaminated by BPA and its analogues or by other contaminants supplied through food.

At the cellular level, we observed increased THC values in clams fed for 7 days with BPAF- and BPF-exposed microalgae, whereas THC was reduced in clams fed for 14 days with BPS-exposed microalgae. Cell proliferation significantly increased in clams fed for 7 days with MIX-exposed microalgae and in those fed for 14 days with BPS-exposed microalgae. Our results highlighted a negative relationship between THC and cell proliferation, both after 7 and 14 days of clam diet (Pearson correlation coefficient: −0.808, *p* < 0.001), with increased THC values generally corresponding to reduced haemocyte proliferation, and vice versa. We hypothesised that the increase in cell proliferation was, at least in part, an attempt of clams to cope with the reduction in THC values, as in the cases of clams fed for 7 days with MIX-exposed microalgae and in those fed for 14 days with BPS-treated microalgae. In contrast, there was no increase in cell proliferation in clams where there were high levels of THC, as in the case of clams fed with BPAF- and BPF-treated microalgae. In our previous study, no significant alterations in THC were observed in clams exposed to the three bisphenols dissolved in water at the same concentrations used in this study, whereas there was a general reduction in both the diameter and volume of haemocytes [15]. In that study, a significant increase in cell proliferation was recorded in clams exposed for 7 and 14 days to the bisphenol mixture. An impairment of THC was also reported by Tang et al. [35] in the clam *Tegillarca granosa* exposed to BPA. Indeed, the authors reported that THC was reduced after 2 weeks of exposure to 10 and 100 ng/L of BPA, with a decreased percentage of red granulocytes and an increased percentage of both basophil granulocytes and hyalinocytes [35]. BPA was also able to reduce THC values in the crab *Charybdis japonica* exposed for 1, 3, and 6 days to 1 mg/L of BPA [36]. In a recent study, the marine bivalve *Lithophaga lithophaga* was exposed for 28 days to 0.25, 1, 2, and 5 µg/L BPA [37]. In that study, the authors observed an increase in THC value in mussels exposed to 0.25, 2 and 5 µg/L. Interestingly, they also observed a reduction in both mean haemocyte diameter and haemocyte nucleus diameter in all the treatments and all the haemocyte cell types (agranulocytes, hyalinocytes, and granulocytes) [37].

Our findings indicate that BPA analogues can affect THC in clams fed with exposed microalgae, like what was observed for BPA in different model species and experimental designs.

Based on the results of the LDH assay, in the present study, we can state that BPA analogues were not able to cause cytotoxic effects in clams fed with exposed microalgae, similar to what was observed in our previous survey with clams exposed via seawater to the same contaminants [15]. However, it has been demonstrated that higher concentrations of both BPF and BPS than those tested in our studies (0, 15.63, 31.25, 62.50, 125, 250, and 500 μM) can cause cytotoxic effects in hepatocytes of the rainbow trout *Oncorhyncus mykiss* after 24 h of treatment [38,39]. As for hydrolytic enzymes, CFH acid phosphatase activity was significantly increased in clams fed for 14 days with BPAF-exposed microalgae, while HL alkaline phosphatase was reduced in clams fed for 7 days with BPAF, BPS and MIX-exposed microalgae and increased in clams after 14-day diet with BPF-exposed microalgae. These results contrast with the findings obtained in clams exposed to BPA analogue-exposed seawater. Indeed, in that case, acid phosphatase activity decreased significantly in CFH after 7 days of exposure of clams to BPAF, BPF and BPS, and after 14 days in BPF-, BPS- and MIX-exposed clams [15]. Moreover, it has recently been demonstrated that BPA can alter both acid phosphatase and lysozyme activity in the marine worm *Urechis unicinctus* exposed for 15 days to 0.07, 7 and 700 μg/L [40]. In detail, that study reported that the acid phosphatase activity of the experimental group exposed to the highest concentration initially increased and then decreased. Moreover, the acid phosphatase activity of BPA-exposed groups was significantly higher than that of the control group on days 5 and 15. Regarding the lysozyme activity, it was significantly decreased in the worms exposed to 0.07 μg/L after 0.5, 1, 3 and 5 days, while it was significantly increased after 10 and 15 days [40]. On the contrary, the exposure to both 7 and 700 μg/L caused a significant decrease in lysozyme activity in all the sampling times [40].

Overall, it seems that BPA analogues can exert different effects on clam haemocytes, depending on exposure modality, via seawater or contaminated diet. However, it is difficult to state which of the two modalities is more dangerous for *R. philippinarum* haemocytes because both (the one adopted in this study and that of the study by Fabrello et al. [15]) caused effects on haemocyte parameters. Moreover, in the present study, phosphatases showed an opposite pattern of variation between HL and CFH, suggesting a release of enzymes from haemocytes into CFH. Indeed, an increase in enzyme activity was generally observed in CFH, whereas a decrease was observed in HL samples of clams during the first week.

The BPA analogue-exposed diet was able to alter the total antioxidant capacity in clam gills, where CUPRAC levels increased in bivalves fed for 7 and 14 days with MIX-exposed microalgae. Moreover, gill CAT activity significantly increased in clams fed for 7 and 14 days with microalgae exposed to BPS and BPF. Interestingly, no significant alterations of the cupric reducing antioxidant capacity (=CUPRAC) were recorded in the digestive gland, suggesting that the main diet-mediated toxic effects occurred in gills during the first part of the feeding process. These findings also suggest that BPA analogue-exposed microalgae caused an increase in hydrogen peroxide, inducing a response of CAT in gills. At the same time, no induction of SOD activity was recorded, suggesting the absence of superoxide anion production. However, further studies are necessary to better elucidate the involvement of bisphenols in ROS production.

This evidence is in accordance with our previous results obtained in clams exposed to contaminated seawater [15]. Indeed, in that study, no significant alterations of CUPRAC levels were observed in digestive glans, whereas there was a significant reduction in the total antioxidant capability in gills from clams exposed for 14 days to BPS and MIX. In addition, gill SOD activity increased significantly in animals exposed to BPS (after 14 days) and MIX (after 7 and 14 days), while CAT activity increased following exposure for 7 and 14 days to MIX [15].

The glutathione cycle plays a pivotal role in both restoring the oxidative status inside the cells and detoxifying xenobiotics. Two of the main glutathione cycle-involved enzymes are GR and GST. In the present study, the first one was significantly affected by the ingestion of exposed microalgae in both the gills and digestive glands of clams. Indeed, GR activity was significantly increased in the gills of clams fed for 7 days with MIX-exposed microalgae, while a reduction in GR activity was found in the digestive gland. The increased activity of GR in gills indicates that the glutathione level (GSH) needed to be restored, probably because it was reduced during antioxidant response. In accordance, the CUPRAC results in gills highlighted an increased antioxidant level in the gills of clams exposed to MIX-treated microalgae. Recently, the effects of BPA on a simplified food chain were investigated by Esperanza et al. [41] in which the clams *Corbicula fluminea* were exposed for 30 days to BPA-exposed microalgae, BPA-exposed water or BPA in both microalgae and water. For the preparation of BPA-exposed microalgae, they exposed *Chlamydomonas reinhardtii* cultures for 24 h at 30 mg/L of BPA, while the tested BPA concentration in water was 7.5 mg/L. Like our study, Esperanza et al. [41] measured several biomarkers in clams. CAT, selenium-dependent glutathione peroxidase (GPX) and total GPX activities were significantly increased in the whole tissues, whereas GR activity increased at all the exposure conditions, even if the exposure to exposed microalgae only caused the lowest GR increase. Contrary to what was observed in our study concerning GST results, which did not reveal any alteration, Esperanza et al. [41] observed a significant inhibition of GST activity after exposure to both BPA-exposed water and microalgae. Regarding BPA analogues, very few studies have been conducted on a simplified marine food chain. In *Chlamys farreri*, the effects of exposure via microalgae alone or microalgae + water contaminated with the BPA analogue tetrabromobisphenol A (TBBPA) were assessed [42]. Firstly, the authors exposed the microalgae *Nitzschia closterium f. minutissima* to 400 μg/L of TBBPA for 24 h and then they provided the microalgae to scallops for 10 days. After 0.5, 1, 3, 6 and 10 days of diet, GST activity, as well as glutathione levels, was significantly increased by experimental conditions in both gills and digestive glands. The authors reported that TBBPA also increased SOD activity at almost all the conditions tested, concluding that TBBPA was able to cause oxidative stress in clams [42]. They also reported a significant reduction in microsomal cytochrome P450 in the gills and digestive gland. Similarly, cytochrome b5 values were significantly reduced by all treatments, even if 3 days of water+food-borne exposure caused a significant increase in gills [42].

As for the results of previous studies on the effects of food contaminated by other contaminants, Iummato et al. [43] analysed the biochemical alterations in the golden mussel *Limnoperna fortunei* under dietary glyphosate exposure. Briefly, the green algae *Scenedesmus vacuolatus* was previously exposed to a mixture of commercial formulation of glyphosate (6 mg/L active principle) with the addition of alkyl aryl polyglycol ether surfactant. Then, the algae were used as food for mussels for 4 weeks and the authors measured the activity of SOD, CAT, GST, and alkaline phosphatase, as well as the glutathione (GSH) content after 1, 7, 14, 21 and 28 days of dietary exposure of mussels. They found that mussels fed on glyphosate-exposed microalgae for 28 days showed increased GST activity, whereas alkaline phosphatase activity was significantly increased at 21 and 28 days of dietary exposure. On the contrary, GSH content and CAT and SOD activities did not show significant differences between treated and untreated bivalves [43]. A similar experimental plan was adopted to assess the effect and transfer of other compounds, such as heavy metals, nanoparticles and hydrocarbons [44,45,46,47]. For instance, the effects of benzo(α)pyrene and 7,12-dimethyl benz(α)anthracene on a marine food chain were evaluated at a concentration of 5 ng/L each on the mussels *Mytilus galloprovincialis* that were directly exposed to contaminated seawater and in fishes *Dicentrarchus labrax* that were exposed to contaminated seawater or fed with exposed mussels for 75 days. Benzo(α)pyrene-monooxygenase activity increased in treated shellfish, while ethoxyresorufin-O-deethylase (EROD) activity increased after 20 days in fishes exposed to contaminated seawater or fed with exposed mussels [44]. More recently, Wang et al. [47] assessed the trophic transfer and effects of titanium dioxide nanoparticles (TiO_2_ NPs) from the marine microalga *Nitzschia closterium* to the scallop *Chlamys farreri*. In detail, they exposed the scallop through aqueous exposure or dietary exposure, and they found increased lysosomal membrane permeability, DNA damage, and histopathological effects induced by TiO_2_ NPs, mainly in scallops after aqueous exposure rather than dietary exposure [47]. In another study, the effects of silver nanoparticles (Ag NPs) (soluble or as lactate Ag NPs) at low concentrations (10 μg/L) were evaluated in the bivalve *Scrobicularia plana* exposed for 14 days directly (water) or via the diet (microalgae) [48]. Interestingly, the authors highlighted that the response of oxidative stress biomarkers (CAT, GST, SOD) in the whole soft tissues of bivalves was more important after dietary than water-borne exposure to Ag. In detail, CAT activity significantly increased by both water and dietary Ag, whereas an Ag-contaminated diet caused significantly increased activity of both SOD and GST [48].

We have also evaluated oxidative damage to both lipids and proteins in clams fed with BPA analogue-exposed microalgae. As a result, oxidative damage to proteins (PCC levels) increased significantly only in the digestive gland of clams fed for 14 days with BPS-exposed microalgae. Moreover, LPO increased in clam gills following a diet of 14 days with BPS-exposed microalgae, while in the digestive gland, LPO levels increased significantly after 14 days in clams fed with MIX-exposed microalgae. The finding that BPS- and MIX-exposed microalgae were able to increase oxidative damage in clams suggested that BPS, if provided via food, both alone or in a mixture, can be considered the most harmful BPA analogue among the three tested.

Esperanza et al. [41] reported an increased LPO level in the clams *Corbicula fluminea* exposed for 30 days to BPA-exposed water or BPA-exposed microalgae and water. Overall, the results obtained in this study indicated that BPA analogues can alter the antioxidant system and cause oxidative damage in clams.

Previous studies indicated that bisphenols can cause neurotoxic effects [49,50]. However, we did not observe neurotoxicity in both the present and the previous study [15]. Therefore, we can exclude that BPA analogues are neurotoxic to clams, at least under the experimental conditions tested.

The effects of a contaminated diet on the activity of enzymes involved in epigenetic mechanisms were also evaluated for the first time in clams. We measured the activity of enzymes involved in the addition and remotion of acetyl groups from the histones, which is a well-known histone post-translational modification that can change the regulation of gene expression [51]. Regarding ecotoxicological studies, it has been demonstrated that exposure to chemical compounds can alter epigenetic mechanisms, as reported in zebrafish exposed to several compounds, such as benzo-α pyrene, heavy metals, PFASs and BPA [52,53,54,55,56]. In particular, it has been demonstrated that exposure to BPA induced global transcriptomic changes in zebrafish embryos and larvae with an alteration in the gene expression of histone deacetylases and DNA methyltransferases [57]. Similarly, Gonzalez-Rojo et al. [58] reported that BPA significantly altered the gene expression of histone acetylation-related genes. In detail, they observed that zebrafish males exposed to 2 mg/L of BPA showed alterations in the expression of two histone deacetylase genes in the testes after 21 days of exposure. There was a decrease in gene expression of the *kat6a* gene and, at the same time, an increase in the *hdac4* gene expression level. Interestingly, they also observed that the global H3 histone acetylation in the testes increased after exposure to both 0.1 mg/L and 2 mg/L, while HAT activity in testes nuclear extracts significantly increased after exposure for 21 days to 2 mg/L of BPA. Our results indicated that BPA analogues provided through food can also alter the enzyme activity of both HAT and HDAC. In particular, clams fed for 14 days with MIX-treated microalgae had significantly reduced HAT activity in the digestive gland. In the same tissue, HDAC activity was significantly increased by the same treatment, but after 7 days of diet. Altered enzyme activities could have caused a reduction in the global histone acetylation level in the digestive gland of clams. On the contrary, gill HDAC activity significantly decreased after 7 days under all the treatments, in comparison to the related control, suggesting an increased histone acetylation level. However, the global histone acetylation level was not evaluated in this study.

Regarding bioaccumulation, no detectable concentrations of bisphenols were found in clams after ingestion of exposed microalgae. However, it is reported that diet can be a vehicle for bisphenols between different food chain levels. Indeed, Hu et al. [42] reported that TBBPA was significantly bioaccumulated after both food-borne and water+food-borne exposure to the mollusc *Clamys farreri*. Interestingly, bioaccumulation was observed in gills, digestive glands, muscles and soft tissues after 0.5, 1, 3 and 6 days of exposure. However, the dietary uptake was lower than the direct TBBPA uptake from water [42]. A similar result was reported in *Scrobicularia plana* for both soluble and lactate Ag NPs, in which bioaccumulation was higher after 14 days of water-borne than dietary exposure [48]. Similar conclusions (greater bioaccumulation in clams exposed to seawater compared to those fed contaminated food) can be formulated for this study because in the previous one, we demonstrated that clams exposed to bisphenols through seawater can accumulate such contaminants [15].

In conclusion, our results suggest that a diet of BPA analogue-exposed microalgae can affect important biomarkers in different clam tissues. Indeed, THC, haemocyte proliferation and two important hydrolytic enzymes, acid and alkaline phosphatases, were affected by exposed microalgae, revealing that BPA analogues can alter some immune responses if provided via food. We also observed an increase in CAT activity, suggesting that a BPA analogue-exposed diet exerted toxic effects, mainly in gills, which is the first organ of the feeding process. Nevertheless, GR activity increased in gills and decreased in digestive glands. Oxidative damage was found in both gills and digestive glands, suggesting that BPA analogues can affect important macromolecules when provided to clams through diet. Lastly, our study demonstrated for the first time that BPA analogue-exposed microalgae can alter epigenetic mechanisms in marine invertebrates. No bioaccumulation of BPA analogues was detected in clam soft tissues. Overall, this study demonstrated that a diet of BPA analogue-exposed microalgae can induce significant alterations of some important biological responses of *R. philippinarum*. To the best of our knowledge, this is the first study demonstrating the effects of ingestion of exposed microalgae in the clam *R. philippinarum*, suggesting a potential ecotoxicological risk for the marine food chain, at least at the first levels.

## Data Availability

The data presented are available in this article.

## Supplementary Materials

The following supporting information can be downloaded at: https://www.mdpi.com/article/10.3390/jox14030069/s1, Figure S1: haemocyte diameter, expressed as µm. Different letters indicate significant differences among all treatments. N = 5; Figure S2: haemocyte volume, expressed as pL. Different letters indicate significant differences among all treatments. N = 5; Figure S3: LDH activity expressed, as OD490 nm. Different letters indicate significant differences among all treatments. N = 5; Figure S4: lysozyme activity in HL, expressed as µg lysozyme/mg proteins. Different letters indicate significant differences among all treatments. N = 5; Figure S5: arylsulfatase activity in HL, expressed as µg p-nitrocatechol/h/mg proteins. Different letters indicate significant differences among all treatments. N = 5; Figure S6: acid phosphatase activity in HL, expressed as U/mg proteins. Different letters indicate significant differences among all treatments. N = 5; Figure S7: alkaline phosphatase activity in CFH, expressed as U/mg proteins. Different letters indicate significant differences among all treatments. N = 5; Figure S8: CUPRAC levels in haemolymph, expressed as mM Trolox Eq/mg proteins. Different letters indicate significant differences among all treatments. N = 5; Figure S9: CUPRAC levels in digestive gland, expressed as mM Trolox Eq/mg proteins. Different letters indicate significant differences among all treatments. N = 5; Figure S10: SOD activity in gills, expressed as U SOD/mg proteins. Different letters indicate significant differences among all treatments. N = 5; Figure S11: CAT activity in the digestive gland, expressed as U CAT/mg proteins. Different letters indicate significant differences among all treatments. N = 5; Figure S12: GST activity in the digestive gland, expressed as nmol/min/mg proteins. Different letters indicate significant differences among all treatments. N = 5; Figure S13: PCC levels in gills expressed as nmol/mg proteins. Different letters indicate significant differences among all treatments. N = 5; Figure S14: AChE activity in gills, expressed as nmol/min/mg proteins. Different letters indicate significant differences among all treatments. N = 5; Figure S15: BChE activity in gills, expressed as nmol/min/mg proteins. Different letters indicate significant differences among all treatments. N = 5; Figure S16: HAT activity in gills, expressed as µmol TNB-/mg proteins. Different letters indicate significant differences among all treatments. N = 5.

## References

[1] Almeida S., Raposo A., Almeida-González M., Carrascosa C. (2018). Bisphenol A: Food Exposure and Impact on Human Health. Compr. Rev. Food Sci. Food Saf..

[2] Vandenberg L.N., Hauser R., Marcus M., Olea N., Welshons W.V. (2007). Human Exposure to Bisphenol A (BPA). Reprod. Toxicol..

[3] Bhandari R.K., Deem S.L., Holliday D.K., Jandegian C.M., Kassotis C.D., Nagel S.C., Tillitt D.E., Vom Saal F.S., Rosenfeld C.S. (2015). Effects of the Environmental Estrogenic Contaminants Bisphenol A and 17α-Ethinyl Estradiol on Sexual Development and Adult Behaviors in Aquatic Wildlife Species. Gen. Comp. Endocrinol..

[4] Chen D., Kannan K., Tan H., Zheng Z., Feng Y.-L., Wu Y., Widelka M. (2016). Bisphenol Analogues Other Than BPA: Environmental Occurrence, Human Exposure, and Toxicity—A Review. Environ. Sci. Technol..

[5] Fabrello J., Matozzo V. (2022). Bisphenol Analogs in Aquatic Environments and Their Effects on Marine Species—A Review. J. Mar. Sci. Eng..

[6] Wang Q., Chen M., Shan G., Chen P., Cui S., Yi S., Zhu L. (2017). Bioaccumulation and Biomagnification of Emerging Bisphenol Analogues in Aquatic Organisms from Taihu Lake, China. Sci. Total Environ..

[7] Yamazaki E., Yamashita N., Taniyasu S., Lam J., Lam P.K.S., Moon H.-B., Jeong Y., Kannan P., Achyuthan H., Munuswamy N. (2015). Bisphenol A and Other Bisphenol Analogues Including BPS and BPF in Surface Water Samples from Japan, China, Korea and India. Ecotoxicol. Environ. Saf..

[8] Huang C., Wu L.-H., Liu G.-Q., Shi L., Guo Y. (2018). Occurrence and Ecological Risk Assessment of Eight Endocrine-Disrupting Chemicals in Urban River Water and Sediments of South China. Arch. Environ. Contam. Toxicol..

[9] Zhao X., Qiu W., Zheng Y., Xiong J., Gao C., Hu S. (2019). Occurrence, Distribution, Bioaccumulation, and Ecological Risk of Bisphenol Analogues, Parabens and Their Metabolites in the Pearl River Estuary, South China. Ecotoxicol. Environ. Saf..

[10] Xie J., Zhao N., Zhang Y., Hu H., Zhao M., Jin H. (2022). Occurrence and Partitioning of Bisphenol Analogues, Triclocarban, and Triclosan in Seawater and Sediment from East China Sea. Chemosphere.

[11] Gao Y., Xiao S.-K., Wu Q., Pan C.-G. (2023). Bisphenol Analogues in Water and Sediment from the Beibu Gulf, South China Sea: Occurrence, Partitioning and Risk Assessment. Sci. Total Environ..

[12] NORMAN Ecotoxicology Database. https://www.norman-network.com/nds/ecotox/lowestPnecsIndex.php.

[13] European Commission, Joint Research Centre (2010). Institute for Health and Consumer Protection. Updated European Union Risk Assessment Report: 4,4’-Isopropylidenediphenol (Bisphenol-A): Environment Addendum of February 2008.

[14] Fabrello J., Guidorizzi S., Ciscato M., Battistuzzi M., Moschin E., Dalla Vecchia F., Moro I., Roverso M., Bogialli S., Matozzo V. (2024). Ultrastructural Changes, Pigment Responses and Bioaccumulation in the Microalga *Phaeodactylum tricornutum* Bohlin Exposed to BPA Analogues. Aquat. Toxicol..

[15] Fabrello J., Ciscato M., Munari M., Vecchiatti A., Roverso M., Bogialli S., Matozzo V. (2023). Ecotoxicological Effects and Bioaccumulation of BPA Analogues and Their Mixture in the Clam *Ruditapes philippinarum*. Mar. Environ. Res..

[16] Guillard R.R.L., Smith W.L., Chanley M.H. (1975). Culture of Phytoplankton for Feeding Marine Invertebrates. Culture of Marine Invertebrate Animals: Proceedings—1st Conference on Culture of Marine Invertebrate Animals Greenport.

[17] Hutchinson T.H., Shillabeer N., Winter M.J., Pickford D.B. (2006). Acute and Chronic Effects of Carrier Solvents in Aquatic Organisms: A Critical Review. Aquat. Toxicol..

[18] Kaviraj A., Bhunia F., Saha N.C. (2004). Toxicity of Methanol to Fish, Crustacean, Oligochaete Worm, and Aquatic Ecosystem. Int. J. Toxicol..

[19] Zucker-Franklin D., Grusky G., Yang J.S. (1983). Arylsulfatase in Natural Killer Cells: Its Possible Role in Cytotoxicity. Proc. Natl. Acad. Sci. USA.

[20] Baum H., Dodgson K.S., Spencer B. (1959). The Assay of Arylsulphatases A and B in Human Urine. Clin. Chim. Acta.

[21] Ross N.W., Firth K.J., Wang A., Burka J.F., Johnson S.C. (2000). Changes in Hydrolytic Enzyme Activities of Naïve Atlantic Salmon *Salmo salar* Skin Mucus Due to Infection with the Salmon Louse *Lepeophtheirus salmonis* and Cortisol Implantation. Dis. Aquat. Org..

[22] Apak R., Güçlü K., Özyürek M., Karademir S.E. (2004). Novel Total Antioxidant Capacity Index for Dietary Polyphenols and Vitamins C and E, Using Their Cupric Ion Reducing Capability in the Presence of Neocuproine:  CUPRAC Method. J. Agric. Food Chem..

[23] Crapo J.D., McCord J.M., Fridovich I., Fleischer S., Packer L. (1978). [41] Preparation and Assay of Superioxide Dismutases. Methods in Enzymology.

[24] Aebi H. (1984). Catalase In Vitro. Methods in Enzymology.

[25] Ellman G.L., Courtney K.D., Andres V., Featherstone R.M. (1961). A New and Rapid Colorimetric Determination of Acetylcholinesterase Activity. Biochem. Pharmacol..

[26] Escartín E., Porte C. (1997). The Use of Cholinesterase and Carboxylesterase Activities from *Mytilus galloprovincialis* in Pollution Monitoring. Environ. Toxicol. Chem..

[27] Smith I.K., Vierheller T.L., Thorne C.A. (1988). Assay of Glutathione Reductase in Crude Tissue Homogenates Using 5,5′-Dithiobis(2-Nitrobenzoic Acid). Anal. Biochem..

[28] Habig W.H., Pabst M.J., Jakoby W.B. (1974). Glutathione S Transferases. The First Enzymatic Step in Mercapturic Acid Formation. J. Biol. Chem..

[29] Mecocci P., Fanó G., Fulle S., MacGarvey U., Shinobu L., Polidori M.C., Cherubini A., Vecchiet J., Senin U., Beal M.F. (1999). Age-Dependent Increases in Oxidative Damage to DNA, Lipids, and Proteins in Human Skeletal Muscle. Free Radic. Biol. Med..

[30] Buege J.A., Aust S.D. (1978). Microsomal Lipid Peroxidation. Methods Enzymol..

[31] Damiens G., Gnassia-Barelli M., Loquès F., Roméo M., Salbert V. (2007). Integrated Biomarker Response Index as a Useful Tool for Environmental Assessment Evaluated Using Transplanted Mussels. Chemosphere.

[32] Bradford M.M. (1976). A Rapid and Sensitive Method for the Quantitation of Microgram Quantities of Protein Utilizing the Principle of Protein-Dye Binding. Anal. Biochem..

[33] Foyn H., Thompson P.R., Arnesen T. (2017). DTNB-Based Quantification of In Vitro Enzymatic N-Terminal Acetyltransferase Activity. Methods Mol. Biol..

[34] Yuan Z., Rezai-Zadeh N., Zhang X., Seto E. (2009). Histone Deacetylase Activity Assay. Methods Mol. Biol..

[35] Tang Y., Zhou W., Sun S., Du X., Han Y., Shi W., Liu G. (2020). Immunotoxicity and Neurotoxicity of Bisphenol A and Microplastics Alone or in Combination to a Bivalve Species, *Tegillarca granosa*. Environ. Pollut..

[36] Peng Y.Q., Wang M.J., Chen H.G., Chen J.H., Gao H., Huang H.H. (2018). Immunological Responses in Haemolymph and Histologic Changes in the Hepatopancreas of *Charybdis japonica* (A. Milne-Edwards, 1861) (Decapoda: Brachyura: Portunidae) Exposed to Bisphenol A. J. Crustac. Biol..

[37] Abd Elkader H.-T.A.E., Al-Shami A.S. (2023). Chronic Exposure to Bisphenol A Induces Behavioural, Neurochemical, Histological, and Ultrastructural Alterations in the Ganglia Tissue of the Date Mussels *Lithophaga lithophaga*. Environ. Sci. Pollut. Res. Int..

[38] Aykut H., Kaptaner B. (2021). In Vitro Effects of Bisphenol F on Antioxidant System Indicators in the Isolated Hepatocytes of Rainbow Trout (*Oncorhyncus mykiss*). Mol. Biol. Rep..

[39] Kaptaner B., Yılmaz C., Aykut H., Doğan E., Fidan C., Bostancı M., Yıldız F. (2021). Bisphenol S Leads to Cytotoxicity-Induced Antioxidant Responses and Oxidative Stress in Isolated Rainbow Trout (*Oncorhyncus mykiss*) Hepatocytes. Mol. Biol. Rep..

[40] Ranđelović D., Gajić G., Mutić J., Pavlović P., Mihailović N., Jovanović S. (2024). Nonspecific Immune, Histology and Accumulation of Marine Worm, *Urechis unicinctus* in Response to Bisphenol A (BPA). Ecotoxicol. Environ. Saf..

[41] Esperanza M., Seoane M., Servia M.J., Cid Á. (2020). Effects of Bisphenol A on the Microalga *Chlamydomonas reinhardtii* and the Clam Corbicula Fluminea. Ecotoxicol. Environ. Saf..

[42] Hu F., Pan L., Xiu M., Liu D. (2015). Dietary Accumulation of Tetrabromobisphenol A and Its Effects on the Scallop *Chlamys farreri*. Comp. Biochem. Physiol. Part C Toxicol. Pharmacol..

[43] Iummato M.M., Sabatini S.E., Cacciatore L.C., Cochón A.C., Cataldo D., de Molina M.D.C.R., Juárez Á.B. (2018). Biochemical Responses of the Golden Mussel *Limnoperna fortunei* under Dietary Glyphosate Exposure. Ecotoxicol. Environ. Saf..

[44] D’adamo R., Pelosi S., Trotta P., Sansone G. (1997). Bioaccumulation and Biomagnification of Polycyclic Aromatic Hydrocarbons in Aquatic Organisms. Mar. Chem..

[45] Wikfors G.H., Twarog J.W., Ferris G.E., Smith B.C., Ukeles R. (1994). Survival and Growth of Post-Set Oysters and Clams on Diets of Cadmium-Contaminated Microalgal Cultures. Mar. Environ. Res..

[46] Ettajani B., Berthet J.C., Amiard H. (2001). Determination of Cadmium Partitioning in Microalgae and Oysters: Contribution to the Assessment of Trophic Transfer. Arch. Environ. Contam. Toxicol..

[47] Wang Z., Xia B., Chen B., Sun X., Zhu L., Zhao J., Du P., Xing B. (2017). Trophic Transfer of TiO2 Nanoparticles from Marine Microalga (*Nitzschia closterium*) to Scallop (*Chlamys farreri*) and Related Toxicity. Environ. Sci. Nano.

[48] Buffet P.E., Pan J.F., Poirier L., Amiard-Triquet C., Amiard J.C., Gaudin P., Risso-de Faverney C., Guibbolini M., Gilliland D., Valsami-Jones E. (2013). Biochemical and Behavioural Responses of the Endobenthic Bivalve *Scrobicularia plana* to Silver Nanoparticles in Seawater and Microalgal Food. Ecotoxicol. Environ. Saf..

[49] Jenzri M., Gharred C., Bouraoui Z., Guerbej H., Jebali J., Gharred T. (2023). Assessment of Single and Combined Effects of Bisphenol-A and Its Analogue Bisphenol-S on Biochemical and Histopathological Responses of Sea Cucumber *Holothuria poli*. Mar. Environ. Res..

[50] Minier C., Forget-Leray J., Bjørnstad A., Camus L. (2008). Multixenobiotic Resistance, Acetyl-Choline Esterase Activity and Total Oxyradical Scavenging Capacity of the Arctic Spider Crab, *Hyas Araneus*, following Exposure to Bisphenol A, Tetra Bromo Diphenyl Ether and Diallyl Phthalate. Mar. Pollut. Bull..

[51] Kouzarides T. (2007). Chromatin Modifications and Their Function. Cell.

[52] Bouwmeester M.C., Ruiter S., Lommelaars T., Sippel J., Hodemaekers H.M., van den Brandhof E.J., Pennings J.L., Kamstra J.H., Jelinek J., Issa J.P.J. (2016). Zebrafish Embryos as a Screen for DNA Methylation Modifications after Compound Exposure. Toxicol. Appl. Pharmacol..

[53] Laing L.V., Viana J., Dempster E.L., Trznadel M., Trunkfield L.A., Webster T.M.U., van Aerle R., Paull G.C., Wilson R.J., Mill J. (2016). Bisphenol A Causes Reproductive Toxicity, Decreases Dnmt1 Transcription, and Reduces Global DNA Methylation in Breeding Zebrafish (*Danio rerio*). Epigenetics.

[54] Corrales J., Fang X., Thornton C., Mei W., Barbazuk W.B., Duke M., Scheffler B.E., Willett K.L. (2014). Effects on Specific Promoter DNA Methylation in Zebrafish Embryos and Larvae following Benzo[a]Pyrene Exposure. Comp. Biochem. Physiol. Part C Toxicol. Pharmacol..

[55] Meyer D.N., Crofts E.J., Akemann C., Gurdziel K., Farr R., Baker B.B., Weber D., Baker T.R. (2020). Developmental Exposure to Pb^2+^ Induces Transgenerational Changes to Zebrafish Brain Transcriptome. Chemosphere.

[56] Khazaee M., Guardian M.G.E., Aga D.S., Ng C.A. (2020). Impacts of Sex and Exposure Duration on Gene Expression in Zebrafish following Perfluorooctane Sulfonate Exposure. Environ. Toxicol. Chem..

[57] Huang W., Zheng S., Wang X., Cai Z., Xiao J., Liu C., Wu K.A. (2020). Transcriptomics-Based Analysis of Toxicity Mechanisms of Zebrafish Embryos and Larvae following Parental Bisphenol A Exposure. Ecotoxicol. Environ. Saf..

[58] González-Rojo S., Lombó M., Fernández-Díez C., Herráez M.P. (2019). Male Exposure to Bisphenol a Impairs Spermatogenesis and Triggers Histone Hyperacetylation in Zebrafish Testes. Environ. Pollut..

