# Supplementary material for: Assessing the Effects of a Diet of BPA Analogue-Exposed Microalgae in the Clam Ruditapes philippinarum
Source: J Xenobiot. 2024 Sep 6;14(3):1221–37. doi: 10.3390/jox14030069 (PMC11417738; doi:10.3390/jox14030069)
Supplement: Supplementary file 1 [file jox-14-00069-s001.zip › jox-3149591-supplementary.pdf]

Assessing the Effects of a Diet of BPA Analogues-Exposed Microalgae in the Clam *Ruditapes philippinarum*

Jacopo Fabrello, Michela Dalla Fontana, Noemi Gaiani, Maria Ciscato, Marco Roverso, Sara Bogialli and Valerio Matozzo

Supplementary Material

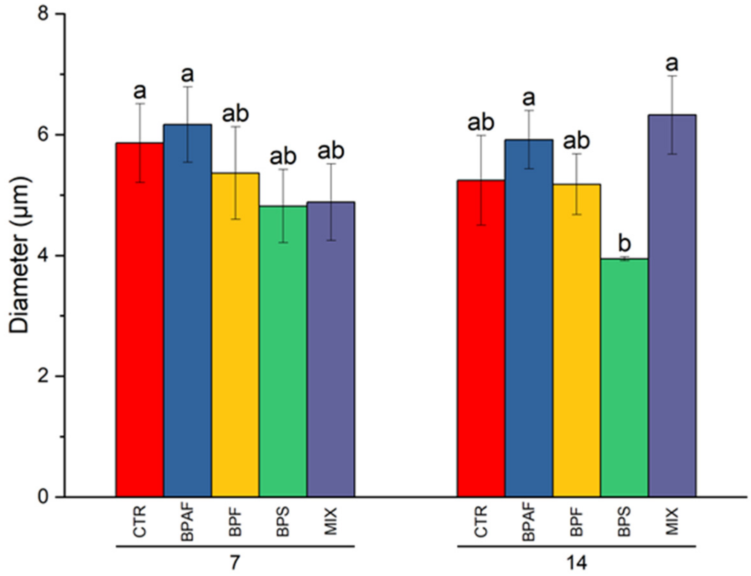

Figure S1: haemocyte diameter, expressed as µm. Different letters indicate significant differences among all treatments. N=5.

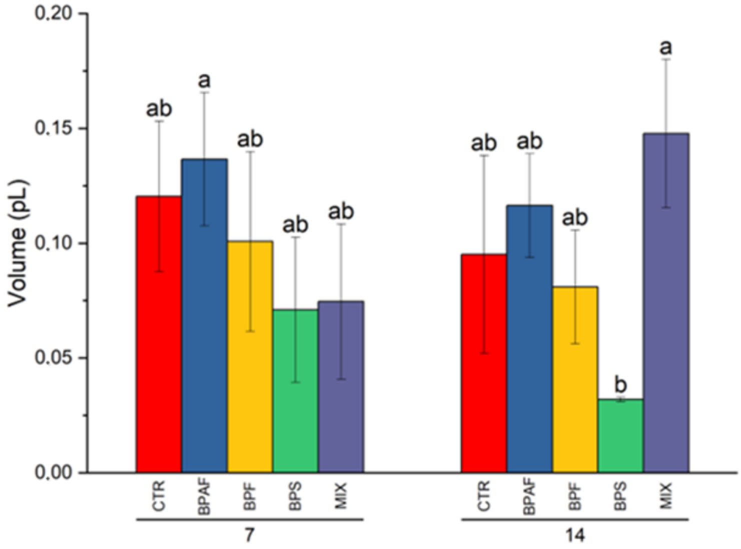

Figure S2: haemocyte volume, expressed as pL. Different letters indicate significant differences among all treatments. N=5.

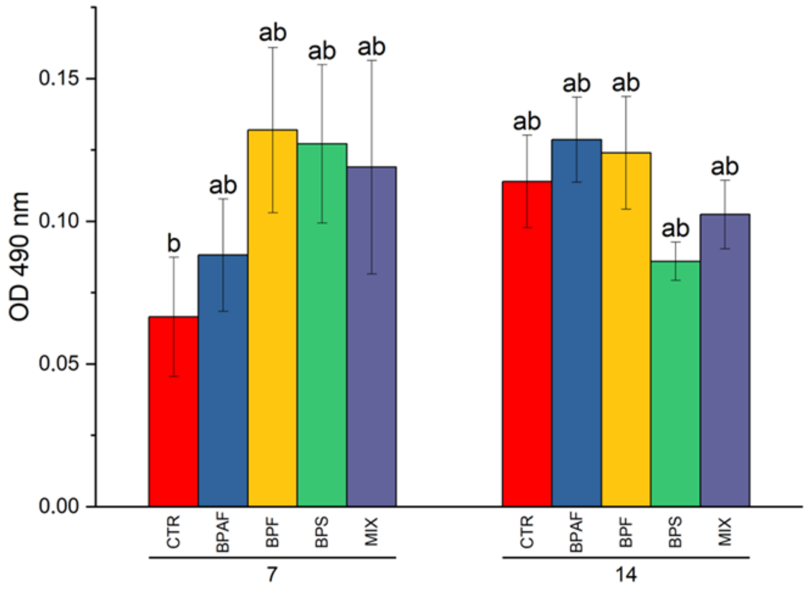

Figure S3: LDH activity expressed, as OD_{490} nm. Different letters indicate significant differences among all treatments. N=5.

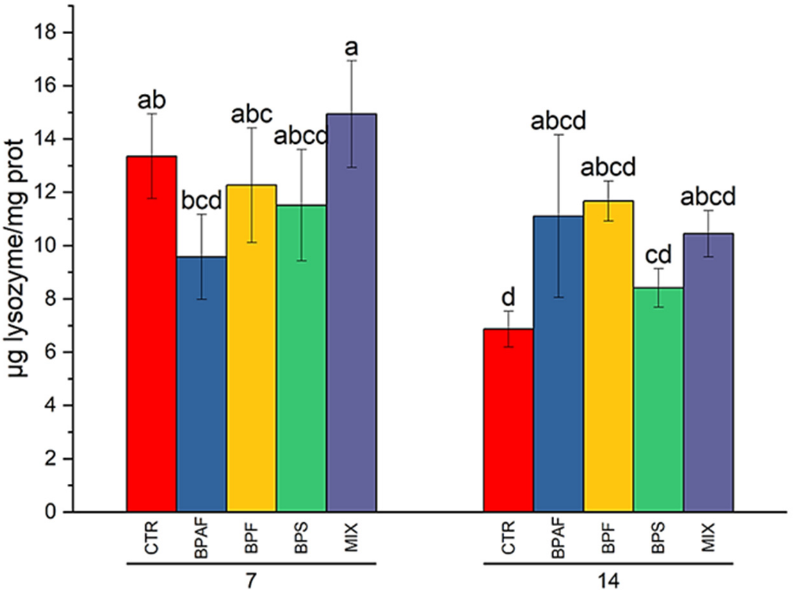

Figure S4: lysozyme activty in HL, expressed as µg lysozyme/mg protein. Different letters indicate significant differences among all treatments. N=5.

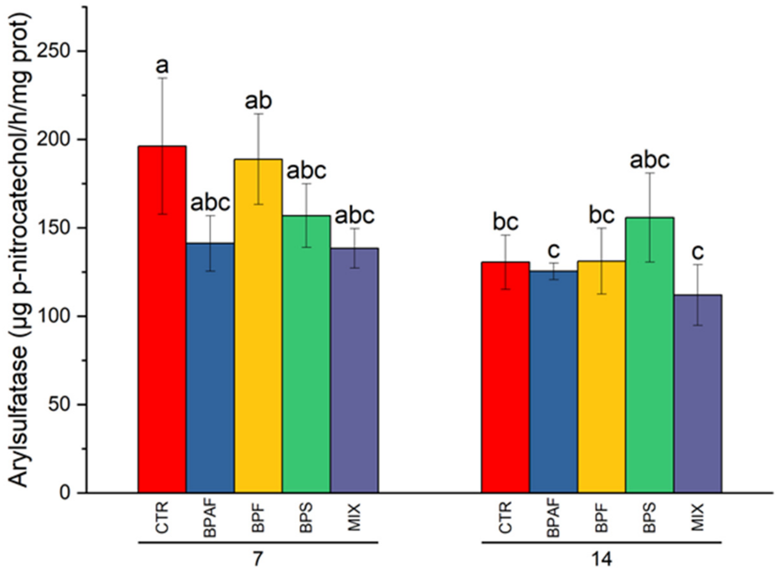

Figure S5: arylsulfatase activity in HL, expressed as µg p-nitrocatechol/h/mg protein. Different letters indicate significant differences among all treatments. N=5.

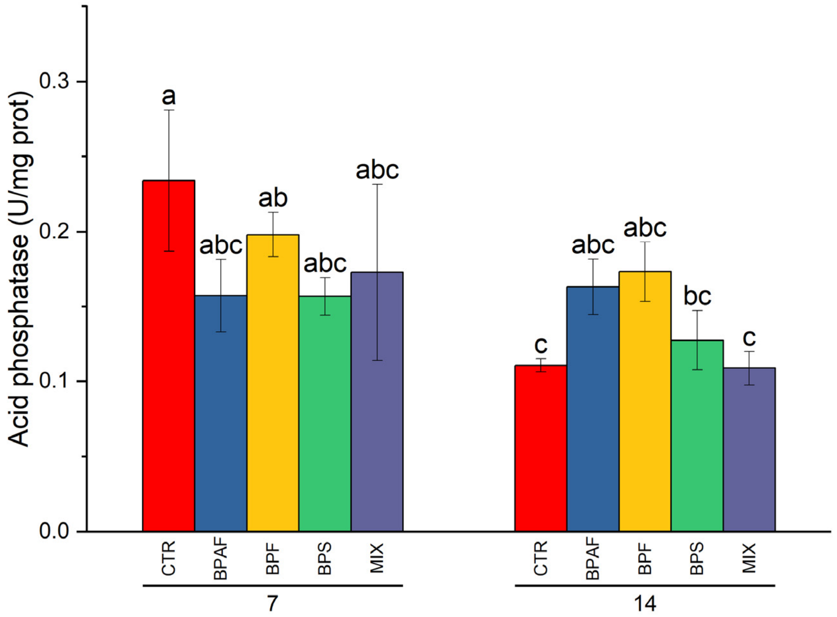

Figure S6: acid phosphatase activity in HL, expressed as U/mg protein. Different letters indicate significant differences among all treatments. N=5.

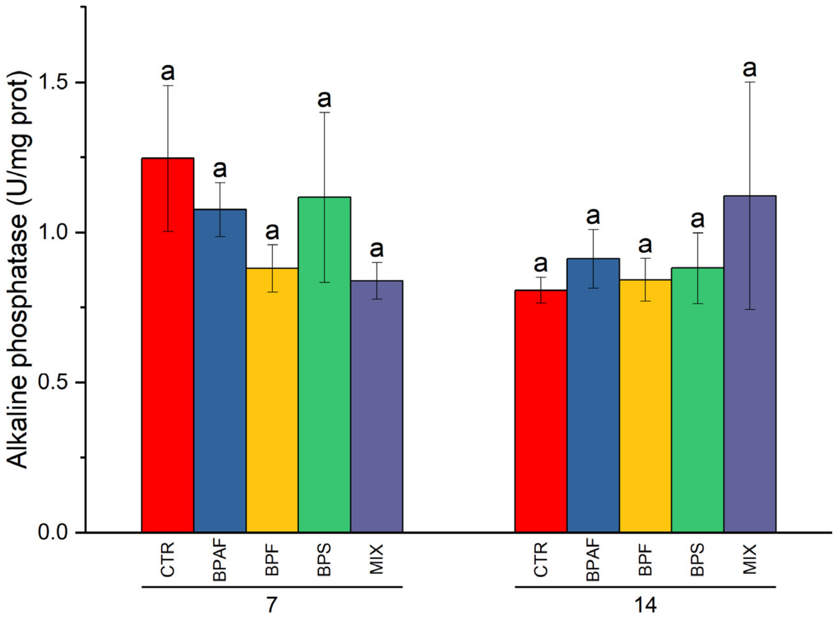

Figure S7: alkaline phosphatase activity in CFH, expressed as U/mg protein. Different letters indicate significant differences among all treatments. N=5.

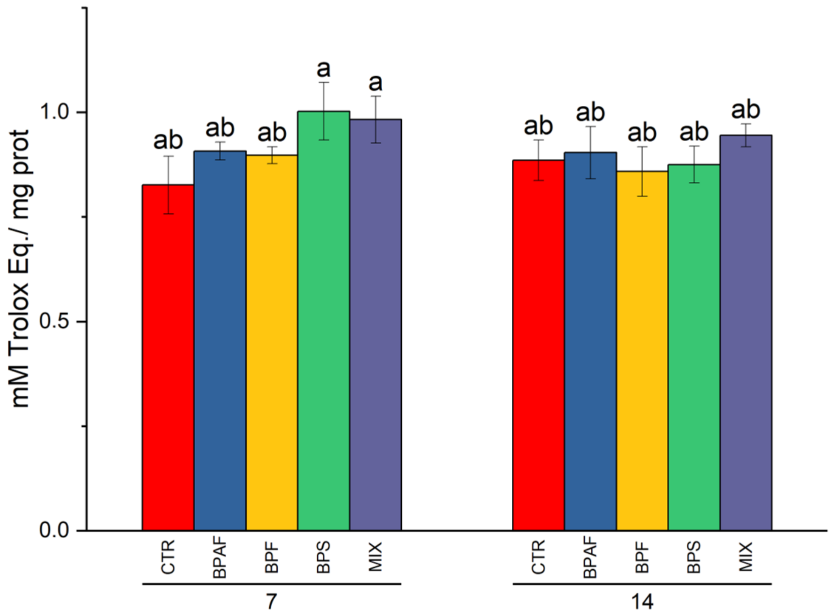

Figure S8: CUPRAC levels in haemolymph, expressed as mM Trolox Eq/mg protein. Different letters indicate significant differences among all treatments. N=5.

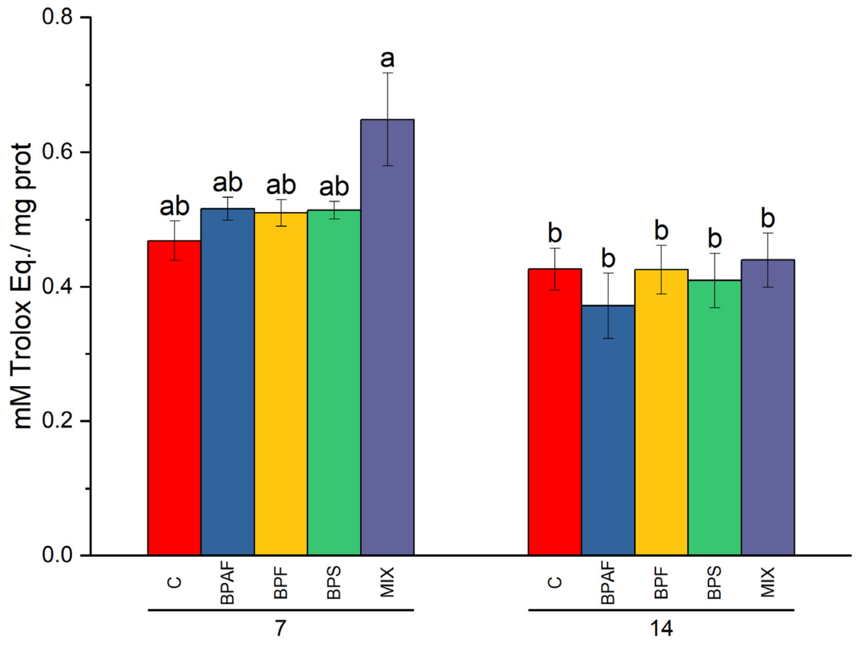

Figure S9: CUPRAC levels in digestive gland, expressed as mM Trolox Eq/mg protein. Different letters indicate significant differences among all treatments. N=5.

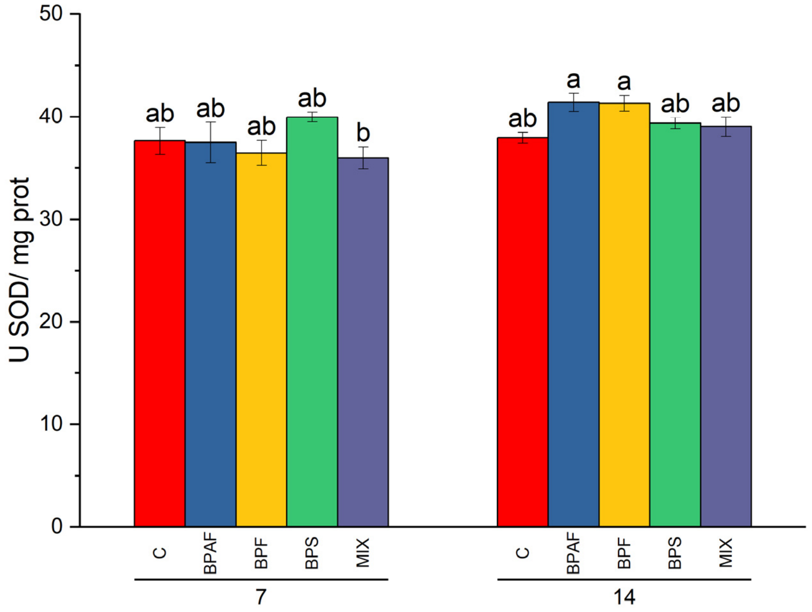

Figure S10: SOD activity in gills, expressed as U SOD/mg protein. Different letters indicate significant differences among all treatments. N=5.

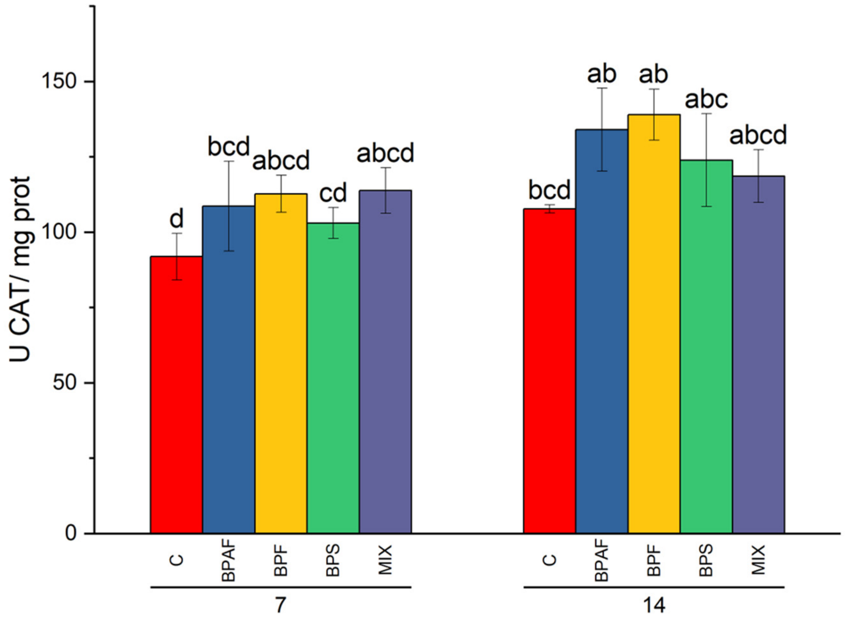

Figure S11: CAT activity in the digestive gland, expressed as U CAT/mg protein. Different letters indicate significant differences among all treatments. N=5.

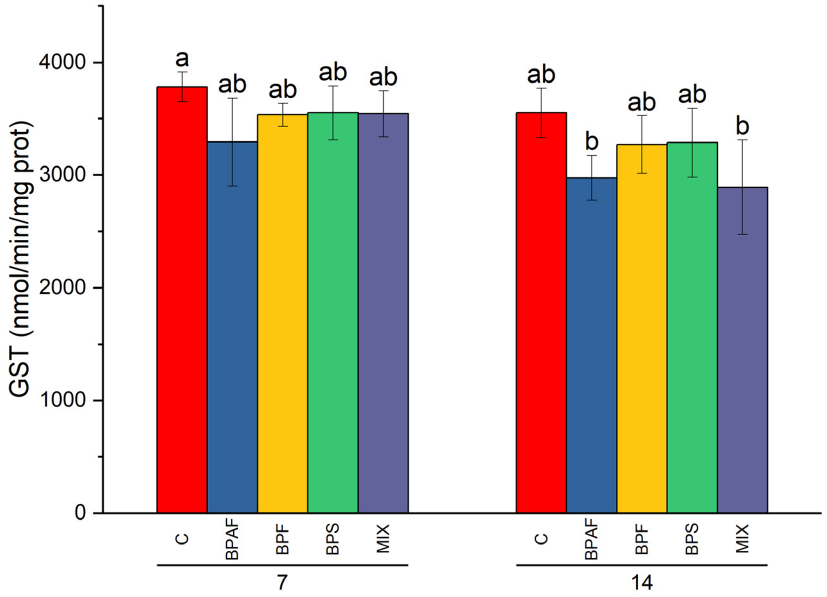

Figure S12: GST activity in the digestive gland, expressed as nmol/min/mg protein. Different letters indicate significant differences among all treatments. N=5.

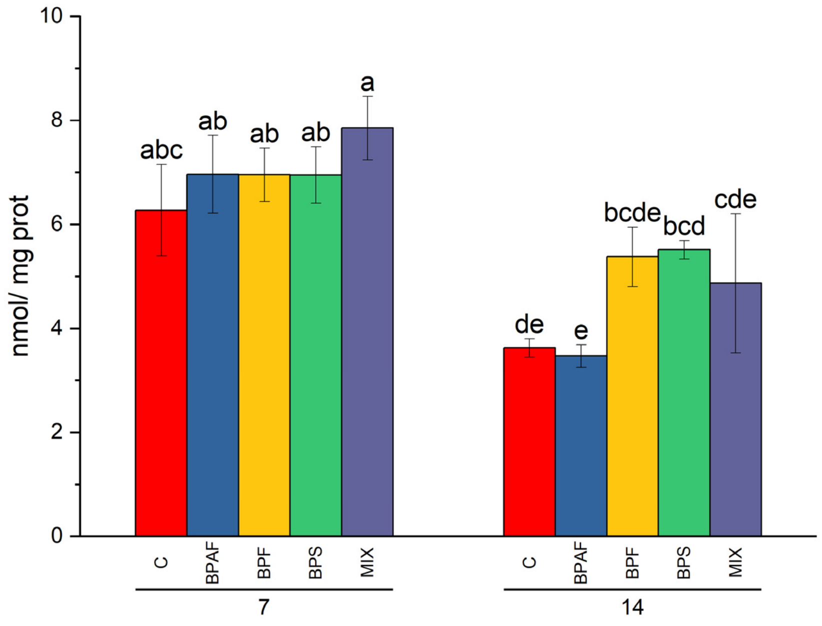

Figure S13: PCC levels in gills expressed as nmol/mg protein. Different letters indicate significant differences among all treatments. N=5.

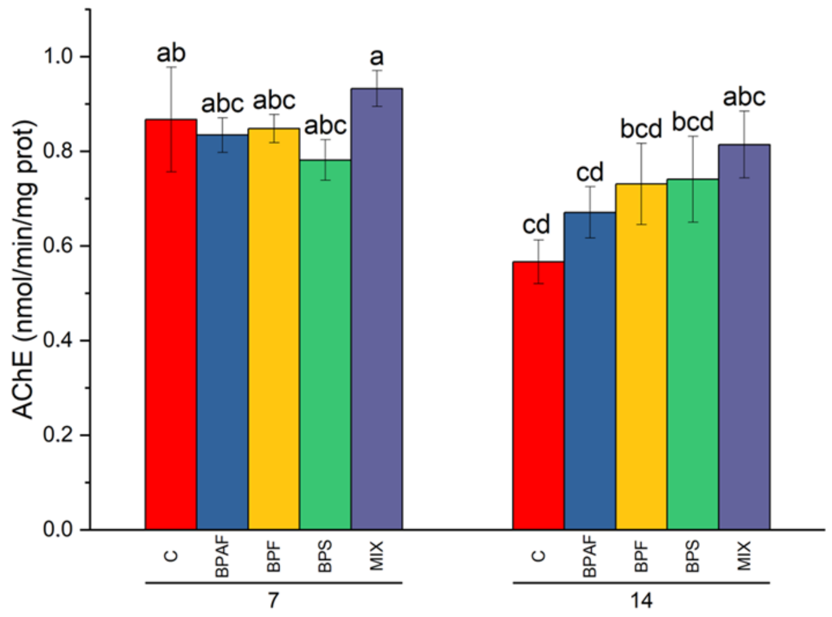

Figure S14: AChE activity in gills, expressed as nmol/min/mg protein. Different letters indicate significant differences among all treatments. N=5.

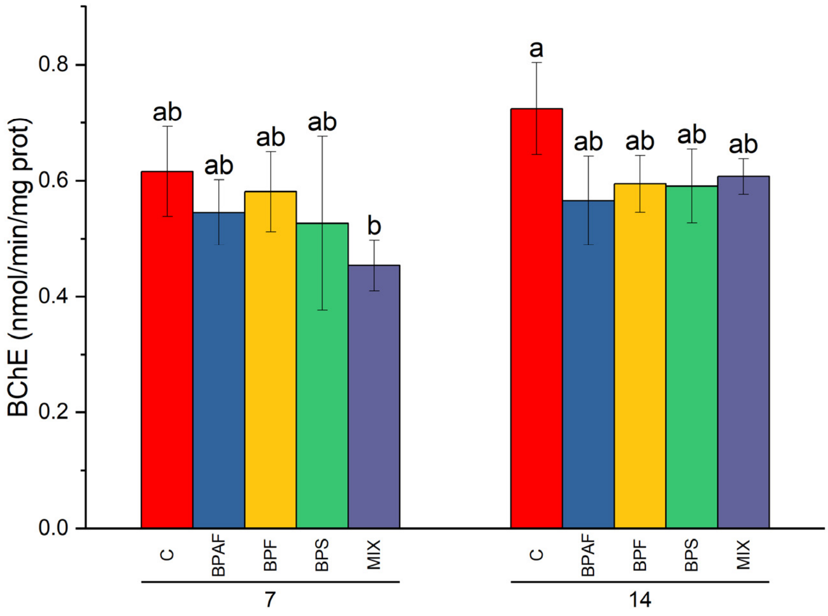

Figure S15: BChE activity in gills, expressed as nmol/min/mg protein. Different letters indicate significant differences among all treatments. N=5.

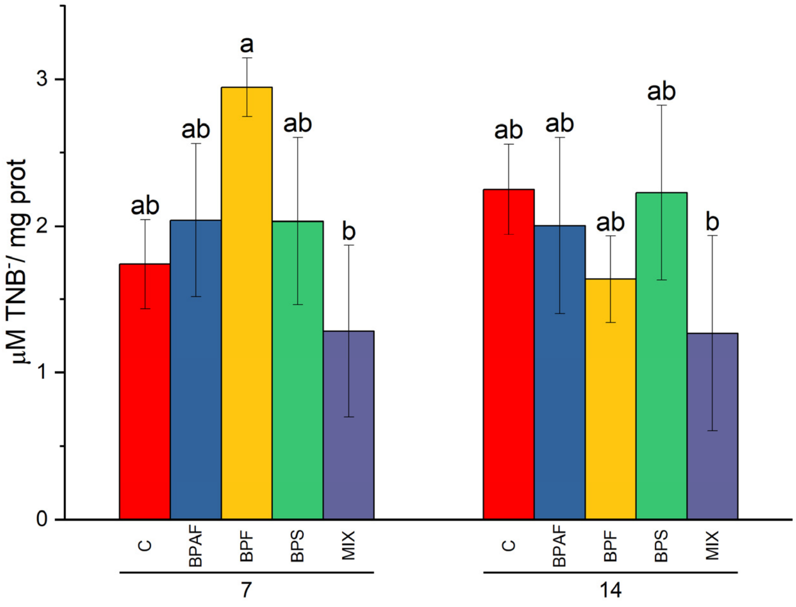

Figure S16: HAT activity in gills, expressed as μmol TNB^-/mg protein. Different letters indicate significant differences among all treatments. N=5.
